# Fulminant Diabetes in a Patient with Advanced Melanoma on Nivolumab

**DOI:** 10.1155/2018/8981375

**Published:** 2018-01-28

**Authors:** Nora Chokr, Hafsa Farooq, Elizabeth Guadalupe

**Affiliations:** ^1^Department of Internal Medicine, Yale School of Medicine, New Haven, CT, USA; ^2^Waterbury Hospital, Waterbury, CT, USA

## Abstract

**Background:**

Anti-PD-1 agents were approved for advanced melanoma after the landmark trial Checkmate-037. Anti-PD-1 agents can breach immunologic tolerance. Fulminant diabetes is an immune endocrinopathy that results from a violent immune attack leading to complete destruction of pancreatic beta cells in genetically predisposed people. We present a rare case of fulminant diabetes precipitated by anti-PD-1 immunotherapy.

**Case:**

A 61-year-old male with advanced melanoma presented with a three-day history of nausea, vomiting, and malaise. He was started on nivolumab and ipilimumab. After the third dose, he developed a generalized rash and was prescribed high-dose prednisone. Labs revealed potassium 9.5 mmol/L, sodium 127 mmol/L, bicarbonate <10 mmol/L, blood glucose 1211 mg/dL, anion gap >31 mmol, arterial blood pH 7.14, and beta-hydroxybutyrate 13.7 mmol/L. He was diagnosed with diabetic ketoacidosis. Hemoglobin A1C was 6.9%. C-peptide was undetectable (<0.1 ng/ml). Glutamic acid decarboxylase autoantibodies, zinc transporter 8 autoantibodies, insulin autoantibodies, islet antigen 2 autoantibodies, and islet cell antibodies were all negative.

**Conclusion:**

Anti-PD-1 immunotherapy is effective in cancers refractory to standard chemotherapy. These agents can precipitate autoimmune disorders. As the use of anti-PD-1 agents is expected to rise, physicians should be educated about the potential side effects. We recommend conducting routine blood glucose checks in patients on these agents.

## 1. Background

Programmed cell death receptor (PD-1) and programmed cell death ligand (PD-L1) were discovered in the 1990s. PD-1/PD-1L checkpoint is involved in immunologic tolerance by regulating T cells at the level of the peripheral tissues. Tumors can express PD-L1 and use these ligands to evade the host's immune system, making this checkpoint a potential target for cancer therapy [1]. This pathway was used to develop monoclonal antibodies that block the interaction between PD-1 receptor and PD-L1 ligand to help restore anticancer immune responses. In 2005, the PD-1/PD-L1 interaction was used to treat animal tumors. Many clinical trials were launched in humans after that. The efficiency of those agents has been shown in tumors belonging to 9 organ systems [2]. They have proven to be very effective in tumors refractory to standard chemotherapy regimens.

The first human trial was conducted in 39 patients with different types of solid cancers who received the fully human IgG4 anti-PD-1 antibody nivolumab (Opdivo®, Bristol-Myers Squibb). Durable responses were observed especially in melanoma, nonsmall cell lung cancer, and renal cell carcinoma [1, 3]. More patients were later enrolled in several clinical trials, some of which are still ongoing. Pembrolizumab (Keytruda®, Merck) was the first anti-PD-1 inhibitor that was approved by the US Food and Drug Administration (FDA) in September 2014 for treating patients with advanced melanoma who had responded poorly to BRAF inhibitors and ipilimumab (Yervoy®, Bristol-Myers Squibb), a monoclonal antibody that upregulates and activates the immune system by targeting CTLA-4 protein. Nivolumab was approved by the FDA on December 22, 2014, for unresectable or metastatic melanoma that progressed after ipilimumab therapy and for patients with positive *BRAF* V600 mutation who failed treatment with BRAF inhibitors. The approval came after the landmark clinical trial Checkmate-037 in which 370 patients with advanced melanoma carrying the BRAF mutation and who failed therapy with ipilimumab and BRAF inhibitors were enrolled and randomized to receive either nivolumab or investigator choice of chemotherapy (dacarbazine or carboplatin plus paclitaxel). The effect of nivolumab was evaluated in the first 120 patients who received the drug and in those who were followed for a minimum duration of 6 months. The overall response rate was 32%, with 4 patients achieving complete response and the rest achieving partial responses. The most common adverse reactions described in the Checkmate-037 trial occurring in more than 10% of the patients were rash, pruritus, cough, upper respiratory infections, and peripheral edema [4]. Other clinical trials followed evaluating the efficacy of anti-PD-1 agents in other types of solid tumors. In March 2015, nivolumab was approved for the treatment of metastatic nonsmall cell lung cancer. In November 2015, nivolumab was approved for metastatic renal cell carcinoma. In May 2016, approval was extended for refractory Hodgkin's lymphoma. In February 2017, nivolumab was approved for locally advanced and metastatic urothelial cancers. These agents have gained popularity since 2014, and over 1 year, FDA has expanded the approval of anti-PD-1 agents across variable cancer types. Currently, clinical trials are studying the role of PD-1 blockage in myelodysplastic syndromes and other hematologic malignancies. The use of checkpoint inhibitors is expected to rise dramatically as we learn more about their efficacy across other types of malignancies. While these medications have proven to be very efficacious in fighting refractory cancers, they are not harmless. Some of the adverse effects are mild and easily controlled; however, some can be very serious and fatal. It is imperative for physicians to be educated about the potential adverse effects of anti-PD-1 immunotherapy. In their attempt to augment the immune response, anti-PD-1 agents can breach immunologic tolerance by upregulating autoreactive T cells. Some of the side effects described in the literature are immune-mediated rash, pneumonitis, colitis, thyroiditis, hepatitis, nephritis, uveitis, adrenalitis, facial nerve paresis, hypophysitis, aseptic meningitis, and fulminant diabetes (FD). FD was first described by Imagawa in Japan. It is a subtype of autoimmune type 1 diabetes that results from a violent and abrupt immune attack leading to a complete destruction of pancreatic beta cells in genetically predisposed people. FD is characterized by an abrupt ketoacidosis that presents with severe hyperglycemia but rather low hemoglobin A1C, low or undetectable C-peptide levels, flu-like symptoms, and elevated pancreatic enzymes [5]. FD has been associated with HLA DRB1^∗^04:05-DQB1^∗^04:01 [6]. It is prevalent in East Asian populations and accounts for a significant proportion of acute onset autoimmune diabetes. After the introduction of anti-PD-1 immunotherapy, FD has become a more commonly encountered phenomenon in the Western world. In this paper, we present a case of FD in a patient with advanced melanoma who was started on the anti-PD-1 agent nivolumab.

## 2. Case Presentation

A 61-year-old male with a history of melanoma metastatic to the chest wall and lungs presents to the emergency department with a three-day history of malaise, nausea, vomiting, polyuria, decreased PO intake, and dizziness. He has no other history of chronic diseases. He was recently started on nivolumab and ipilimumab, a combination of anti-PD-1 and anti-CTLA-4 immunotherapy. After the third dose, he developed a generalized maculopapular rash, a known side effect of his immunotherapy regimen, for which he was started on high-dose prednisone three days prior to presentation. The patient reports antecedent fatigue and nausea that profoundly worsened after starting high-dose prednisone. On presentation, he appeared in distress. He was tachycardic with a heart rate of 126 beats per minute, tachypneic with a respiratory rate of 24 breaths per minute, and hypotensive with a blood pressure of 90/50 mmHg. His oral mucosa was dry. His labs revealed potassium 9.5 mmol/L, sodium 127 mmol/L, bicarbonate <10 mmol/L, blood glucose 1211 mg/dL, anion gap >31 mmol, arterial blood pH 7.14, beta-hydroxybutyrate 13.7 mmol/L, lactic acid 2.4 mmol/L, serum creatinine 4.55, positive urine ketones, urine glucose >1000 mg/dL, and serum lipase 414 IU/L. The patient was admitted to the intensive care unit for management of diabetic ketoacidosis (DKA). He has no prior history of diabetes. He was managed with IV hydration and insulin drip and later transitioned to subcutaneous insulin after the anion gap closed. He required daily insulin adjustments, and it was hard to achieve optimal glycemic control. During his hospitalization, workup revealed a hemoglobin A1C of 6.9%. His serum C-peptide was undetectable <0.1 ng/ml. Glutamic acid decarboxylase autoantibodies (GADA) and zinc transporter 8 autoantibodies (ZnT8A) analyzed by ELISA, insulin autoantibodies (IAA) analyzed by radioimmunoassay, islet antigen 2 autoantibodies (IA-2A) analyzed by radiobinding assay, and islet cell antibodies (ICA) analyzed by immunofluorescence assay were all negative. To date, the patient is on subcutaneous basal-bolus insulin regimen with glargine and lispro, respectively. Our patient did not carry the high-risk HLA haplotype of FD identified in Japanese populations.

## 3. Discussion

Our patient is one of a handful of cases described in the literature of FD caused by anti-PD-1 cancer therapy. In FD, patients present with severe hyperglycemia or diabetic ketoacidosis; however, they have unexpectedly low hemoglobin A1C levels which are probably due to the abrupt onset of this endocrinopathy. C-peptide levels, like in our patient, are low or undetectable. Islet cell autoantibodies are undetectable which suggest that beta cells are completely destroyed via a process that is not entirely similar to the pathophysiology of the classically known autoimmune type 1 diabetes. In FD, the islet cells are attacked by autoreactive T cells. FD was initially thought to be an entirely cell-mediated phenomenon; however, some patients described in the literature tested positive for 1 or more islet cell autoantibodies either at disease onset or later, which suggests that a humoral immune response is also implicated in the pathophysiology of FD (Table 1). FD was first described in the literature in the setting of anti-PD-1 immunotherapy in early 2015, shortly after the drugs were approved by the FDA. Patients who develop FD become insulin dependent for life. It can manifest 1 week to a few months after starting the therapy. Diagnosis should be prompt because of the increased risk of death within the first 24 hours. It is worth mentioning that these drugs can upset the gastrointestinal tract and cause nausea, vomiting, and abdominal pain that can all be managed symptomatically. It is important to distinguish these drug adverse effects from DKA symptoms [5]. Our patient must have had high blood glucose levels days to weeks prior to presentation. The high-dose prednisone that he was started on for the rash definitely played a major role in exacerbating his condition.

There are around 19 cases described to date of fulminant diabetes in 6 male and 13 female patients after receiving anti-PD-1 agents with either nivolumab or pembrolizumab, with or without other chemotherapeutic drugs, for different types of advanced cancers like melanoma, small and nonsmall cell lung cancer, renal cell carcinoma, and Hodgkin's lymphoma. The mean age is 59.7 years. Fifteen of these patients presented with diabetic ketoacidosis, and four with new onset hyperglycemia, with or without ketonuria, within weeks to months following treatment onset. The mean hemoglobin A1C was 7.7%. C-peptide levels in all cases were either undetectable or in the low normal range on presentation but eventually became undetectable. Two patients tested positive for 3 out of 4 islet antibodies, nine patients tested positive for GADA only, and the rest had negative antibody titers. Few of these patients had HLA haplotypes associated with the development of autoimmune type 1 diabetes. These cases illustrate that anti-PD-1 agents affect the T cells regulatory pathways triggering both humoral and cell-mediated autoimmunity [7] (Table 1).

## 4. Conclusion

Anti-PD-1 immunotherapy has shown great efficacy in refractory cancers. PD-1/PD-1L is a complex pathway involved in autoimmunity, and many of the details of its molecular signaling remain unclear to date. This complexity accounts for the selective emergence of autoimmune diseases in some patients but not others [3]. Although there are no formal guidelines for managing the adverse effects of anti-PD-1 immunotherapy, most of these are managed with high-dose steroids. This can be critical in autoimmune diabetes. Hyperglycemia with no ketoacidosis can be a “silent” adverse drug reaction, because it is not felt or seen like, for example, colitis and rash, respectively. We recommend obtaining baseline hemoglobin A1C levels and conducting routine blood glucose checks in patients on anti-PD-1 agents, especially in those presenting with other drug reactions that require high-dose steroids. This can help detect early hyperglycemia and prevent fulminant diabetes, which can be fatal, if not managed promptly.

## Figures and Tables

**Table 1:** Characteristics of patients reported in the literature so far with fulminant diabetes from anti-PD-1 immunotherapy.

Study	Age (years)/sex	Cancer diagnosis	Anti-PD-1 agent	Presentation	HbA1C (%)	C-peptide	Antibody positivity
Hughes et al. [7]	55/F	Melanoma	Nivolumab	DKA	6.9	Undetectable	No
	83/F	NSCLC	Nivolumab	DKA	7.7	Undetectable	GADA
	63/M	RCC	Nivolumab	Hyperglycemia	8.2	Low	GADA, IAA, ICA
	58/M	SCLC	Nivolumab	DKA	9.7	Undetectable	GADA
	64/F	Melanoma	Pembrolizumab	Hyperglycemia	7.4	Low	No
Martin-Liberal et al. [8]	54/F	Melanoma	Pembrolizumab	DKA	NA	NA	GADA
Mellati et al. [9]	70/M	NSCLC	Nivolumab	DKA	9.8	Low	NA
	66/F	Jaw sarcomatoid squamous cell carcinoma	NA	DKA	9.4	Undetectable	GADA
Gaudy et al. [10]	44/F	Melanoma	Pembrolizumab	DKA	6.85	Undetectable	GADA
Okamoto et al. [11]	55/F	Melanoma	Nivolumab	Hyperglycemia	7	Undetectable	No
Miyoshi et al. [5]	66/F	Melanoma	Nivolumab	DKA	7.3	Undetectable	No
Lowe et al. [12]	54/F	Melanoma	Nivolumab	DKA	NA	Undetectable	GADA
Munakata et al. [13]	71/M	Hodgkin lymphoma	Nivolumab	Hyperglycemia	7.3	Undetectable	No
Ishikawa et al. [14]	54/F	Melanoma	Nivolumab	DKA	7	Low	No
Li et al. [15]	63/F	NSCLC	Nivolumab	DKA	<6.4%	NA	GADA
Araújo et al. [16]	73/F	NSCLC	Nivolumab	DKA	7.2	Undetectable	GADA
Alzenaidi et al. 2017 [17]	47/M	Melanoma	Nivolumab	DKA	8	Undetectable	GADA
Godwin et al. [18]	34/F	NSCLC	Nivolumab	DKA	7.1	Undetectable	GADA, IA-2, IAA
This study	61/M	Melanoma	Nivolumab	DKA	6.9	Undetectable	No

DKA: diabetic ketoacidosis; F: female; NSCLC: nonsmall cell lung cancer; SCLC: small cell lung cancer; RCC: renal cell carcinoma; GADA: glutamic acid decarboxylase autoantibodies; IAA: insulin autoantibodies; ICA: islet cell antibodies; NA: not available; IA-2: islet 2 autoantibody.

## Abbreviations

DKA: Diabetic ketoacidosis
ICU: Intensive care unit
PD-1: Antiprogrammed death 1 receptor
PD-1L: Programmed death ligand
GADA: Glutamic acid decarboxylase autoantibodies
IAA: Insulin autoantibodies
IA-2A: Islet antigen 2 autoantibodies
ICA: Islet cell antibodies
ZnT8A: Zinc transporter 8 autoantibodies.
